# Integrated analysis of ultra-deep proteomes in cortex, cerebrospinal fluid and serum reveals a mitochondrial signature in Alzheimer’s disease

**DOI:** 10.1186/s13024-020-00384-6

**Published:** 2020-07-25

**Authors:** Hong Wang, Kaushik Kumar Dey, Ping-Chung Chen, Yuxin Li, Mingming Niu, Ji-Hoon Cho, Xusheng Wang, Bing Bai, Yun Jiao, Surendhar Reddy Chepyala, Vahram Haroutunian, Bin Zhang, Thomas G. Beach, Junmin Peng

**Affiliations:** 1 Departments of Structural Biology and Developmental Neurobiology, St. Jude Children’s Research Hospital, Memphis, TN 38105 USA; 2 Center for Proteomics and Metabolomics, St. Jude Children’s Research Hospital, Memphis, TN 38105 USA; 3 Present address: Department of Biology, University of North Dakota, Grand Forks, ND 58202 USA; 4 Present address: Department of Laboratory Medicine, Nanjing Drum Tower Hospital, Nanjing University Medical School, Nanjing, 210008 Jiangsu China; 5 Departments of Psychiatry and Neuroscience, The Alzheimer’s Disease Research Center, Icahn School of Medicine at Mount Sinai, New York, NY 10029 USA; 6 Mental Illness Research, Education and Clinical Center (MIRECC), James J. Peters VA Medical Center, Bronx, NY 10468 USA; 7 Department of Genetics and Genomic Sciences and Department of Pharmacological Sciences, Mount Sinai Center for Transformative Disease Modeling, Icahn Institute for Data Science and Genomic Technology, Icahn School of Medicine at Mount Sinai, New York, NY 10029 USA; 8 Banner Sun Health Research Institute, Sun City, AZ 85351 USA

**Keywords:** Alzheimer’s disease, Biomarker, Cerebrospinal fluid, Brain tissue, Cortex, Blood, Plasma, Serum, Mass spectrometry, Proteomics, Proteome, Tandem mass tag, Systems biology

## Abstract

**Background:**

Based on amyloid cascade and tau hypotheses, protein biomarkers of different Aβ and tau species in cerebrospinal fluid (CSF) and blood/plasma/serum have been examined to correlate with brain pathology. Recently, unbiased proteomic profiling of these human samples has been initiated to identify a large number of novel AD biomarker candidates, but it is challenging to define reliable candidates for subsequent large-scale validation.

**Methods:**

We present a comprehensive strategy to identify biomarker candidates of high confidence by integrating multiple proteomes in AD, including cortex, CSF and serum. The proteomes were analyzed by the multiplexed tandem-mass-tag (TMT) method, extensive liquid chromatography (LC) fractionation and high-resolution tandem mass spectrometry (MS/MS) for ultra-deep coverage. A systems biology approach was used to prioritize the most promising AD signature proteins from all proteomic datasets. Finally, candidate biomarkers identified by the MS discovery were validated by the enzyme-linked immunosorbent (ELISA) and TOMAHAQ targeted MS assays.

**Results:**

We quantified 13,833, 5941, and 4826 proteins from human cortex, CSF and serum, respectively. Compared to other studies, we analyzed a total of 10 proteomic datasets, covering 17,541 proteins (13,216 genes) in 365 AD, mild cognitive impairment (MCI) and control cases. Our ultra-deep CSF profiling of 20 cases uncovered the majority of previously reported AD biomarker candidates, most of which, however, displayed no statistical significance except SMOC1 and TGFB2. Interestingly, the AD CSF showed evident decrease of a large number of mitochondria proteins that were only detectable in our ultra-deep analysis. Further integration of 4 cortex and 4 CSF cohort proteomes highlighted 6 CSF biomarkers (SMOC1, C1QTNF5, OLFML3, SLIT2, SPON1, and GPNMB) that were consistently identified in at least 2 independent datasets. We also profiled CSF in the 5xFAD mouse model to validate amyloidosis-induced changes, and found consistent mitochondrial decreases (SOD2, PRDX3, ALDH6A1, ETFB, HADHA, and CYB5R3) in both human and mouse samples. In addition, comparison of cortex and serum led to an AD-correlated protein panel of CTHRC1, GFAP and OLFM3. In summary, 37 proteins emerged as potential AD signatures across cortex, CSF and serum, and strikingly, 59% of these were mitochondria proteins, emphasizing mitochondrial dysfunction in AD. Selected biomarker candidates were further validated by ELISA and TOMAHAQ assays. Finally, we prioritized the most promising AD signature proteins including SMOC1, TAU, GFAP, SUCLG2, PRDX3, and NTN1 by integrating all proteomic datasets.

**Conclusions:**

Our results demonstrate that novel AD biomarker candidates are identified and confirmed by proteomic studies of brain tissue and biofluids, providing a rich resource for large-scale biomarker validation for the AD community.

## Background

Alzheimer’s disease (AD), the most common cause of dementia, affects more than 5 million Americans and an estimated 47 million worldwide [1]. It is a progressive neurodegenerative brain disorder clinically characterized by extracellular amyloid plaques deposition, intracellular neurofibrillary tangle growth, memory and cognition impairments [2–4]. Traditionally, AD is diagnosed by patient’s symptoms, memory and behavior tests, and confirmed by post-mortem brain pathologies, with recent additions of brain imaging of these pathologies [5]. According to the amyloid cascade and tau hypotheses, protein biomarkers in cerebrospinal fluid (CSF) and blood/plasma/serum have also been developed or under development, including amyloid-β (Aβ) level, Aβ42/Aβ40 ratio, total tau level, and the accumulation of phosphorylated tau isoform [6–9]. Currently, techniques like structural magnetic resonance imaging (MRI), and molecular imaging of deposited Aβ and tau proteins using positron emission tomography (PET), are highly accurate in detecting the presence of pathophysiological and neuropathological changes of AD and are used in the drug development [10, 11]. But their high cost and insufficient accessibility are being major limitations [12]. Therefore, the field will benefit from increasing availability of blood-related and CSF biomarkers that systematically reflect the AD pathogenesis. To accomplish this ambitious goal, unbiased profiling of human CSF and blood samples has been attempted to reveal AD novel biomarkers to improve diagnosis and prognosis [13].

Proteomic profiling of human specimens is largely achieved by the approach of modern mass spectrometry (MS) [14, 15]. With the advances in peptide separation power by multi-dimensional liquid chromatography (LC), and the improvement of MS resolution and scan rate, MS can profile more than 12,000 proteins (> 10,000 genes) from mammalian tissue samples [16–18]. Both data dependent acquisition strategy (e.g. label free method and stable isotope labeling) [19] and data-independent acquisition strategy [20] are currently used. Tandem-mass-tag (TMT) has been emerging as a common stable isotope labeling method [21], enabling up to 16-plexed analysis [22]. Although ratio compression occurs during quantification because of peptide co-elution, the limitation can be addressed by the introduction of the MS3 method [23], extensive LC fractionation, MS optimization, and computational correction [24], to allow deep proteomic analysis [18, 25, 26].

Compared with the analysis of human cell cultures or solid tissues, comprehensive proteomic analysis of human CSF and blood is often difficult because individual protein concentration spans a large dynamic range of at least 10 orders of magnitude [27]. For example, albumin is the most abundant protein in human blood present at a concentration of ~ 50 mg/ml. In sharp contrast, the cytokine of interleukin-6 is detected at a concentration of 4.2 pg/ml in healthy individuals [28, 29]. To reduce protein dynamic range, antibody-based depletion of the most abundant proteins is often utilized to enhance the detection of proteins of low abundance [30–32], but the depletion is not complete and can introduce experimental variations [32]. More recently, our group utilized the superior separation capacity of the latest TMT-LC/LC-MS/MS to bypass the depletion step and detect about 5000 proteins in human CSF and serum toward AD biomarker discovery [18, 25]. In addition, several other groups used the similar strategies in AD biomarker studies [33, 34]. Although a large number of new protein biomarker candidates have been reported by the MS analysis, it is not straightforward to determine the reliable candidates for the following large-scale validation studies.

Here we introduce an integrated approach to analyze unbiased, large-scale and ultra-deep proteomes in cortex, CSF and serum from multiple independent cohorts, totaling 10 large proteomic datasets with 17,541 proteins (13,216 genes) from 365 samples. We also analyzed CSF samples from an AD mouse model to show protein correlation with amyloidosis. Given this urgent requirement for biomarkers that reflect AD neuropathology, comprehensive systems-based approaches are likely to develop network-based biomarkers across multiple human tissues (brain cortex and biofluids). Our ultra-deep CSF profiling uncovered the majority of previously reported AD CSF biomarkers and also identified deregulated proteins associated with mitochondrial function. Further assimilation of human cortex and CSF proteomes and validation in the mouse model show amyloidosis-induced changes. Finally, we introduced a comprehensive systems approach to prioritize the most promising targets for Alzheimer’s disease.

## Materials and methods

### Human brain cortex, cerebrospinal fluid, and serum

Human brain cortex, CSF, and serum specimen were provided by the brain and body donation program at Banner Sun Health Research Institute and the Alzheimer’s Disease Research Center at Icahn School of Medicine at Mount Sinai with well-established criteria for clinical and pathological diagnoses [35, 36]. All subjects consented to the study. A total of 110 human brain tissue, 20 CSF, and 11 serum cases were used as discovery cohorts (datasets i, ii, v, x in Table 1) for the present proteomics study. All samples were frozen and stored at − 80 °C in aliquots of polyethylene tubes until use. Sample information is provided in Table 1.

**Table 1:** Summary of Human and Mouse Proteome Datasets for Biomarker Analysis

Tissue Type	Dataset	Total Case	AD	MCI	Control	Proteins Quantified	Reference
Human Cortex	i	48	19	7	22	12,578	This study and cohort 1 in Bai B, et al. Neuron. 2020
Human Cortex	ii	62	23	0	39	13,702	Cohort 2 in Bai B, et al. Neuron. 2020
Human Cortex	iii	40	10	20	10	8817	Cortex cohort 1 in Higginbotham L, bioRxiv. 2019
Human Cortex	iv	27	9	8	10	11,244	Cortex cohort 2 in Higginbotham L, bioRxiv. 2019
Human CSF	v	20	11	0	9	5941	This study and Bai B, et al. Neuron. 2020
Human CSF	vi	40	20	0	20	2875	CSF cohort 1 in Higginbotham L, bioRxiv. 2019
Human CSF	vii	96	33	31	32	792	CSF cohort 2 in Higginbotham L, bioRxiv. 2019
Human CSF	viii	10	5	0	5	2321	Sathe G, et al. Proteomics Clinical Applications. 2019
^a^ Mouse CSF	ix	11	6	0	5	1058	This study
Human Serum	x	11	6	0	5	4826	Dey KK, et al. Clinical Proteomics. 2019
**Summary**	**10**	**365**	**142**	**66**	**157**	**17,541**	

^a^Note: AD cases are 5 x FAD mice, control cases are age matched healthy mice

### Mouse cerebrospinal fluid

Wide type (WT) control and 5xFAD transgenic mice that overexpress familial AD mutants (the Swedish mutation, K670N/M671L; the Florida mutation, I716V; and the London mutation, V717I) and PS1 (M146L, L286V) transgenes at the age of 9–12 months were used for the spinal fluid collection. Mice were bred and maintained in a specific pathogen free facility in the Animal Resource Center at St. Jude Children’s Research Hospital. All protocols were approved by the Institutional Animal Care and Use Committee. CSF samples were collected following an established protocol [37], and then were snap-frozen in liquid nitrogen, and stored at − 80 °C before analysis.

### Protein extraction and quantification

The frozen samples were lysed in the fresh lysis buffer comprised of 50 mM HEPES, pH 8.5, 8 M urea, and 0.5% sodium deoxycholate with 1x phosphatase inhibitor cocktail (PhosSTOP, Sigma-Aldrich). Protein extraction and concentration measurement were done by our established protocol [25, 38]. In brief, BCA assay (Thermo Fisher Scientific) was used for measuring protein amount, and the quantifications were further confirmed by short SDS Coomassie-stained gel [39]. The protein lysates were stored at − 80 °C in aliquots before use.

### Protein digestion and tandem-mass-tag (TMT) labeling

Protein digestion and labeling were carried out with a previously optimized protocol [38, 40]. ~ 0.1 mg of quantified proteins in the lysis buffer with 8 M urea were first digested with Lys-C (Wako, 1:100 w/w) at 21 °C for 2 h, and then the solution was diluted 4-fold to urea concentration of 2 M; trypsin (Promega, 1:50 w/w) was further added for digestion at 21 °C for overnight. The digestion process was terminated by 1% trifluoroacetic acid (TFA). The supernatant was desalted with Sep-Pak C18 cartridge (Waters), and then dried by a speed vacuum. Each sample was re-dissolved in 50 mM HEPES (pH 8.5) for TMT reaction for 30 min, and then mixed and pooled equally. Pooled samples were desalted for the subsequent fractionation by offline basic pH Liquid chromatography (LC).

### Extensive two-dimensional LC/LC-MS/MS analysis

The pooled TMT labeled peptides were resolved and fractionated by offline basic pH reverse phase LC, and each of the fractions was analyzed by acidic pH reverse phase LC coupled with MS/MS analysis [24, 41, 42]. The offline basic pH LC was performed with an XBridge C18 column (3.5 μm particle size, 4.6 mm × 25 cm, Waters), buffer A (10 mM ammonium formate, pH 8.0), buffer B (95% acetonitrile, 10 mM ammonium formate, pH 8.0), using a 2–3 h gradient of 15–35% buffer B [38]. Up to 180 fractions were collected every minute for biofluid samples, and a total of 40 concatenated fractions were collected for cortex. In the acidic pH LC-MS/MS analysis, fractions were analyzed sequentially on a column (75 μm × 15–30 cm, 1.9 μm C18 resin from Dr. Maisch GmbH, 65 °C to reduce backpressure) coupled with a Fusion or Q Exactive HF Orbitrap mass spectrometer (Thermo Fisher Scientific). Peptides were analyzed with a 1–3 h gradient (buffer A: 0.2% formic acid, 5% DMSO; buffer B: buffer A plus 65% acetonitrile). For mass spectrometer settings, positive ion mode and data-dependent acquisition were applied with one full MS scan followed by a 20 MS/MS scans. MS1 scans were collected at a resolution of 60,000,1 × 10^6^ AGC and 50 ms maximal ion time*;* higher energy collision-induced dissociation (HCD) was set to 32–38% normalized collision energy; ~ 1.0 m/z isolation window with 0.3 *m/z* offset was applied; MS2 spectra were acquired at a resolution of 60,000, fixed first mass of 120 *m/z*, 410–1600 *m/z*, 1 × 10^5^ AGC, 100–150 ms maximal ion time, and ~ 15 s of dynamic exclusion.

### Protein identification and quantification by the JUMP software suite

The bioinformatics processing of protein identification and quantification were carried out with the JUMP software suite [43–45]. In brief, MS/MS raw data were searched against a target-decoy database to estimate false discovery rate (FDR) [46]. We combined the downloaded Swiss-Prot, TrEMBL, and UCSC databases and removed redundancy (human: 83,955 entries) to create the database. Main search parameters were set at precursor and product ion mass tolerance (±15 ppm), full trypticity, maximal modification sites *(n = 3*), maximal missed cleavage *(n = 2*), static mass shift including carbamidomethyl modification (+ 57.02146 on Cys), TMT tags (+ 229.16293 on Lys and N-termini), and dynamic mass shift for oxidation (+ 15.99491 on Met). Peptide-spectrum matches (PSM) were filtered by mass accuracy, clustered by precursor ion charge, and the cutoffs of JUMP-based matching scores (J-score and ΔJn). The peptide was represented by the protein with the highest PSMs according to the rule of parsimony when one peptide was matched to multiple homologous proteins [47]. Protein quantification was performed based on the reporter ions from MS2 using our previously optimized method [24].

### Differential expression analyses of proteome datasets

Blood contamination is a major established covariate in tissue/biofluids proteome analysis, especially in serum/plasma [48]. Thus we applied a robust linear regression model for blood contamination correction [49]. The residual was then used for the following differential expression analysis except for certain blood covariates (e.g. coagulation in serum proteome) that are biased in AD and control groups in the small cohorts of discovery proteomes. Blood contamination outlier samples were removed when biased blood covariates were detected. For instance, three outlier samples were removed in CSF due to erythrocyte contamination. Differential expression analyses of discovery proteomes were carried out via the LIMMA R package [50], and multiple test correction was performed by Benjamini-Hochberg (BH) procedure [51]. For individual proteome analysis, we applied two cutoffs, including Z score transformed Log_2_ fold change > 2 and FDR < 0.05 or *p* value < 0.05. For multiple proteome integration, Z score difference > 2 and FDR < 0.2 were used.

### Principal component analysis

Principal component analysis (PCA) was used to visualize the differences among different sample groups in discovery proteomes. Log_2_ transformed relative expression of all proteins was used as features of PCA. The pairwise Euclidean distance between features was calculated. PCA was performed using the R package prcomp [52].

### Integrated ranking of proteins in individual datasets though order statistics

To integrate multiple proteome datasets from distinct tissue/biofluids and independent studies to prioritize disease proteins and pathways in AD, a comprehensive order statistics-based protein ranking was carried out similarly as previously described [17, 18], which combined N distinct sets of protein rankings to output one integrated ranking. In brief, a total of 10 individual datasets from three independent deep proteomic studies were integrated for this analysis. The ranks of proteins were normalized by the total number of proteins in each dataset and the integrated protein ranking was generated by the framework of order statistics [53, 54]. Specifically, the ranks of each data source were randomly permutated for 1000 times to derive null Q values, and the empirical *p* values were then derived from the estimated null Q distribution. Multiple test was corrected by BH method. The integration was carried out in a 3-step tiered manner. Discovery cohorts or reference cohorts were first separately consolidated. Proteomes of individual tissue/biofluids were then combined into cortex, CSF, or serum ranking. Finally, after removing proteins without any change in CSF and serum data sets, the three ranks were integrated into a final integrative rank. To summarize the integrated ranking into pathway rankings, we performed pathway enrichment by GSEA [55]. The value and FDR were derived by permuting the proteins sets for 1000 times in a core pathway extracted from GO, KEGG, and Hallmark. Pathways with FDR < 0.05 were accepted as enriched pathways.

### TOMAHAQ targeted MS validation assay

The TOMAHAQ analysis was executed essentially the same as the previous study [25] using an established protocol [56]. The selected AK2 and PCK2 peptides were synthesized, purified, and labeled by a TMT0 reagent from Thermo Fisher Scientific, and were then spiked into the TMT11-labeled pooled samples with optimized quantities. These labeled synthetic and target peptide mixture were analyzed on a Fusion Orbitrap mass spectrometer following the same steps applied in the previous biomarker study [25]. Acquired targeted MS3 level quantification were compared with the original discovery MS analyses. Finally, Pearson correlation between the TOMAHAQ and the discovery MS assays were carried out to confirm the validity of these biomarker candidates.

### ELISA validation assay

GPNMB protein levels in the CSF samples from 7 AD and 7 healthy controls were detected by human Osteoactivin (GPNMB) ELISA kit (RayBiotech, US). CSF samples were diluted 3 folds with the diluent buffer before the assay. ELISA was carried out in accordance with the manufacturer’s manual. Student’s t-test was applied for the DE analysis between AD and Ctl groups, and Pearson correlation was performed to compare the quantification between ELISA and the discovery MS assay.

## Results

### Comprehensive integration of ultra-deep AD proteomes in cortex, CSF and serum

**Fig. 1:**
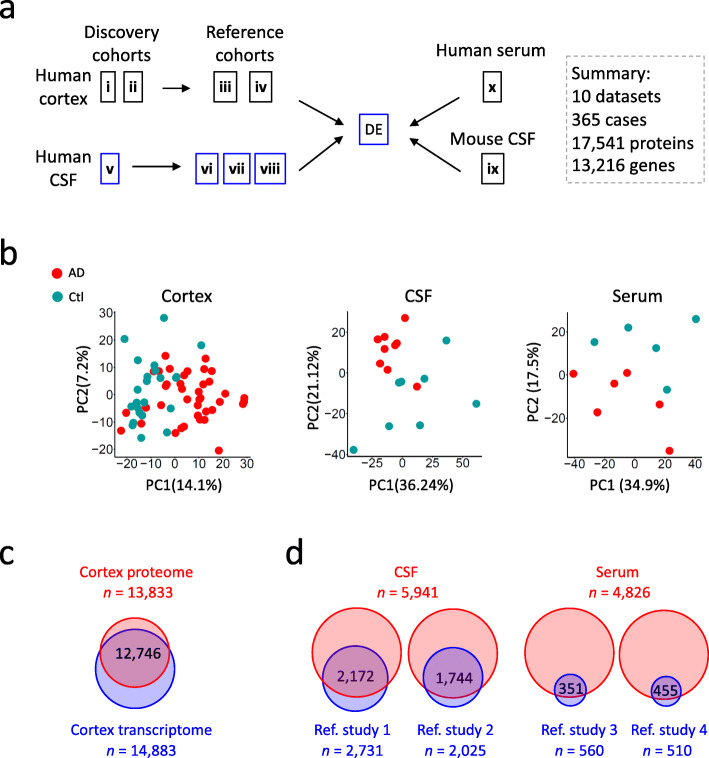
Comprehensive integration of ultra-deep cortex, CSF and serum proteome datasets for biomarker analyses in Alzheimer’s disease. **a** Workflow for data integration. The human brain cortex proteomes consist of 4 datasets including 2 discovery cohorts (data i and ii) that were validated by 2 reference cohorts (data iii and iv). The human cerebrospinal fluid (CSF) proteomes consist of 4 individual datasets including one discovery cohort (data v) that was validated by 3 reference cohorts (data vi, vii, viii). Differential expression (DE) of CSF proteome was carried out through LIMMA R package, and then integrated with cortex proteome. Next, the human CSF proteome was compared with mouse CSF (data ix). Finally, the cortex and CSF proteomes were integrated with the serum proteome (data x). **b** Principle component analyses (PCA) of discovery proteomes. Dot plots show two-dimensional principle component analyses of all quantified proteins in the representative discovery datasets including human cortex (ii), Human CSF (v), and human serum (x). Protein expression values of all datasets were log2-transformed for PCA analyses. **c** Advanced tissue proteome profiling pipeline achieves ultra-deep proteome coverage in cortex. The unique proteins quantified in the cortex discovery cohorts were combined and then compared to the cortex transcriptome with consensus normalized expression (NX) values > 1 in the Human Protein Atlas database. **d** Advanced biofluid proteome profiling platform achieves ultra-deep proteome coverage in human CSF and serum. The CSF proteome was compared to the two deepest MS-based CSF proteome studies in AD so far, Reference study 1 (data vi, Higginbotham L, BioRxiv, 2019) and Reference study 2 (data viii, Sathe G, Proteomics Clin Appl. 2019), similarly the serum proteome data was compared to the two recent MS-based AD serum protein biomarker studies, Reference study 3 (Ashton N, Science Advances, 2019) and Reference study 4 (Lan J, Journal of Proteome Research, 2018)

To systematically investigate AD biofluid biomarkers that are associated with AD pathogenesis, we performed comprehensive integrated analyses of 10 independent AD proteomic datasets covering 5 ultra-deep discovery datasets and 5 deep reference datasets from brain cortex, CSF and serum (Table 1). The cortex proteome consists of 2 discovery cohorts and 2 reference cohorts. The CSF proteome consists of 1 discovery cohort and 3 reference cohorts. The 5 reference datasets were mined from 2 independent biofluid proteome studies of AD [33, 34]. The integrative analyses were carried out via a CSF-centric manner, and datasets were assigned with labels from i to x (Table 1**,** Fig. 1a). In total, we analyzed 17,541 proteins (13,216 genes) from 365 AD, MCI and healthy control cases (Fig. 1a), representing the most comprehensive AD proteomic data to our knowledge.

We first examined the proteomic data quality of our 5 discovery datasets from cortex, CSF and serum (Supplementary Table S1, S2, S3, S4 and S5). It appears that the AD and control samples are distinguishable by principle component analysis of the entire proteomic datasets (Fig. 1b). We next examined the profiling depth of these proteomic datasets. Ultra-deep proteomic profiling was achieved through our newly established pipeline, which combines undepleted biofluid sample processing, multiplexed tandem-mass-tag labeling, extensive two-dimensional liquid chromatography fractionation and high-resolution tandem mass spectrometry (termed TMT-LC/LC-MS/MS) [18, 25]. As a result, our cortex proteome (13,833 proteins from cortex datasets i and ii) can cover 86% of the expressed cortex transcriptome based on the human protein atlas database [57] (Fig. 1c). Our CSF discovery proteome (5941 proteins in dataset v) covers 80 and 86% of reference study 1 (2731 proteins in dataset vi) and reference study 2 (2025 proteins in dataset viii), respectively, while the reference studies 1 and 2 cover only 37 and 29% of our CSF proteome. Similarly, our serum discovery proteome was compared with two recent AD serum proteome studies [58, 59]. Our dataset (4826 proteins in dataset x) covers 63 and 89% of the two reference datasets (560 and 510 proteins), respectively, while the reference datasets cover only 7 and 9% of our proteome (Fig. 1d). Considering the low coverage of the two human serum datasets, we did not use them in our analysis. Together, these comparisons confirm the high quality of our analyzed proteomes, highlighting the deep coverage of the AD tissue/biofluid proteomes.

### Ultra-deep CSF proteome profiling identifies evident mitochondrial protein reduction in Alzheimer’s disease

**Fig. 2:**
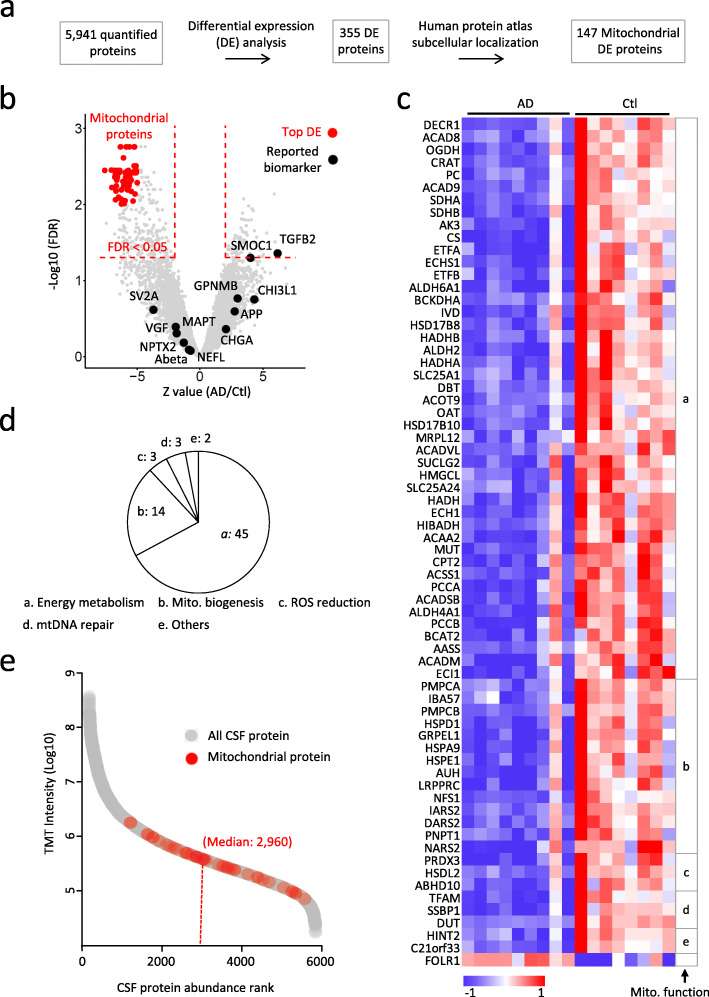
Ultra-deep CSF proteome profiling identifies evident mitochondrial protein reduction in Alzheimer’s disease. **a** Workflow for CSF proteome analysis. **b** Ultra-deep CSF proteome unveiled evident decrease of mitochondrial proteins in AD. The X-axis of the volcano plot for all quantified CSF proteins shows the Z score transformed log2 level fold changes comparing AD to Ctl. Y-axis shows the -log10 level FDR value. Previously reported AD CSF biomarkers are plotted in black. Top DE proteins with FDR < 0.01 and Z value < − 5 are plotted in red. Red dashed lines indicate the DE cutoff of FDR < 0.05 and Z score difference > 2. **c** Majority of top DEs are mitochondrial proteins showing decreased level in AD. Heatmap shows the relative expression of top DE proteins with Z score difference > 5 and FDR < 0.01 comparing AD to Ctl, these DE proteins are classified into distinct groups (**a**-**e**) according to their mitochondrial functions as indicated on the right side of the heatmap. **d** Pie chart shows the mitochondrial functional groups classified in panel c. The number of proteins in each subgroup is labeled. **e** Deep profiling depth is a prerequisite for confident detection of evident mitochondrial protein changes. CSF proteins are plotted as a function of their concentration rank (x-axis) and their mean log10 level TMT intensity in all samples (y-axis). Top DE mitochondrial proteins with Z score difference > 5 and FDR < 0.01 were plotted in red. The median concentration rank of these mitochondrial proteins is labeled and marked by dashed red line

AD CSF biomarkers have been extensively explored in shallow coverage due to technical challenges. Although many biomarkers have been proposed, most of them cannot be reproducible across laboratories; new proteomics techniques that can support in-depth profiling are urgently needed for biomarker studies. To explore novel AD biomarker candidates in an ultra-deep proteome setting, we recently developed a new in-depth biofluid profiling pipeline [25] and applied it to the analysis of 20 CSF samples. In total, we quantified 5941 unique proteins with a false discovery rate (FDR) of 1% in 11 AD and 9 healthy control cases. Three sample outliers were removed due to blood contamination. DE analysis was carried out through LIMMA R package, resulting in 355 DE CSF proteins (Z value of log_2_Ratio > 2 and FDR < 0.05, Fig. 2a). Our ultra-deep CSF proteome identified most of previously reported AD CSF biomarker candidates (12 out of 13, Supplemental Table S6), however, the majority of them displayed no statistical significance except SMOC1 and TGFB2, which may be due to the small sample size in our pilot study and/or the small changes of these proteins in AD (Fig. 2b). Nevertheless, we still observed 68 top DE proteins, even under a highly stringent threshold (Z value > 5 and FDR < 0.01). Remarkably, 67 out of the 68 top DE proteins are mitochondrial proteins (Fig. 2b, c), and most of them are tightly correlated with the others (Fig. S1). These proteins are known to have functional roles in supporting energy metabolism, mitochondrial biogenesis, reactive oxygen species reduction, and mt DNA repair (Fig. 2d).

This correlated mitochondrial protein decrease in AD is striking but was barely reported in previous CSF studies. To understand why these proteins were missed in previous studies, we ranked all quantified proteins according to their abundance, and found that these top DE mitochondrial proteins were presented in the CSF at low abundance, with a median abundance rank of 2960 (Fig. 2e). We then performed systematic investigation of the DE proteins using distinct proteome coverage. If the coverage is as shallow as the depth of 500 proteins, it is sufficient to detect many previously reported AD biomarker candidates but miss all of these top mitochondrial DE proteins. While a small fraction of these mitochondrial proteins starts to show up with the depth of 2000–3000 proteins, the majority of these proteins are revealed with the depth of at least 4000 proteins (Fig. S2). Thus, ultra-deep profiling is a prerequisite to detect these protein changes in AD CSF proteome. In summary, our CSF proteomic analysis covers the most of previously reported AD CSF biomarkers and unveils evident mitochondrial protein reduction in the AD patients.

### Integration of CSF and cortex proteomes discovers consistent CSF biomarkers in Alzheimer’s disease across independent studies

**Fig. 3:**
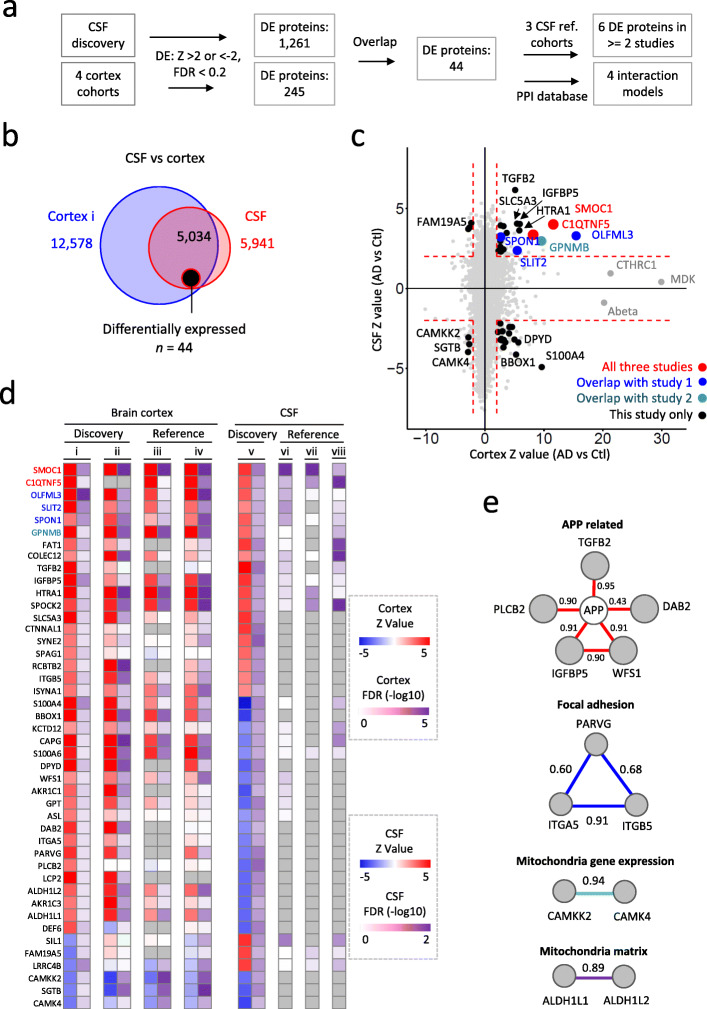
Integrated analysis of cortex and CSF proteomes unveils consistent CSF biomarkers in AD across independent studies. **a** Scheme for the integration of cortex and CSF proteomes. **b** Venn diagram shows overlap of quantified proteins in cortex discovery cohort (data i) and CSF discovery cohort (data v). **c** Integration of cortex and CSF proteomes identifies consistent CSF biomarkers in AD across independent studies. Proteins quantified in both cortex and CSF are plotted as a function of their Z score comparing AD to Ctl in cortex (x-axis) and their Z score comparing AD to Ctl in CSF (y-axis). Fourty-four DE proteins with Z score difference > 2 and FDR < 0.2 in both proteomes are plotted in black. Proteins consistently showed up as AD biomarkers in all three independent CSF studies are labeled in red, proteins stood out in this study and reference study 1 (Higginbotham L, BioRxiv, 2019) are labeled in blue, and proteins emerged in this study and reference study 2 (Sathe G, Proteomics Clin Appl. 2019) are labeled in turquoise. Red dashed lines indicate Z value difference > 2 in CSF and cortex. **d** Heatmap shows Z score transformed log2 level fold changes and -log10 FDR values of the 44 DE proteins comparing AD to control in cortex and CSF proteome datasets. **e** Integration of DE proteins and protein-protein interaction (PPI) database unveils enrichment of amyloid pathology and mitochondrial functions. Protein-protein interaction modules were derived from superimposing the 44 DE proteins along with APP and TAU on STRING PPI database. The interaction modules were build based only on the most confident interaction sources including experiments and database. The default statistic criteria of STRING with a cutoff of minimum interaction score of 0.4 was applied to derive PPI modules. The pairwise PPI interactions scores are displayed next to the edges

To investigate CSF protein changes that are associated with AD pathology, we systematically integrated 4 cohorts of cortex and 4 cohorts of CSF datasets from three independent MS-based proteome profiling studies (Fig. 3a). The cortex proteome covered majority of proteins quantified in the CSF (Fig. 3b). We applied a cutoff (Z value > 2 and FDR < 0.2) for all datasets, resulting in 1261 DE proteins in the CSF and 245 DE proteins in the cortex; 44 out of them were changed in both proteomes (Figs. 3b**-**d), with most of them showing increases in both cortex and CSF (e.g. TGFB2, IGFBP5, and SLC5A3) or increase in the cortex but decrease in CSF (e.g. DPYD and S100A4, similar to the expression pattern of Aβ42 peptide [60]). Interestingly, MDK, CTHRC1, and Aβ, which were reported as the most significantly elevated proteins in our AD brain cortex study [18], were not significantly changed in the small cohort of CSF samples (Fig. 3c). Superimposing these 44 proteins along with APP and TAU onto STRING protein-protein interaction database [61] elucidated 4 protein interaction modules associated with amyloid pathology and mitochondrial functions, while no TAU related protein interaction module was identified with this small list (Fig. 3e). Notably, most of these module proteins are correlated with the amyloid level (Fig. S3).

**Fig. 4:**
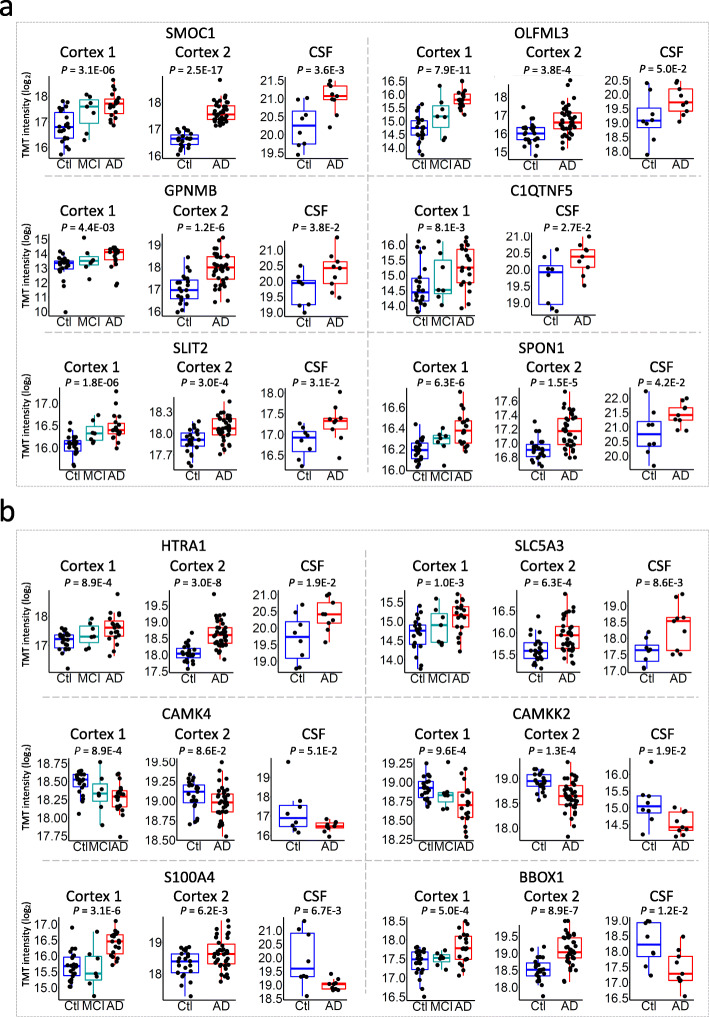
Expression levels of reported and novel AD CSF biomarker candidates in cortex and CSF proteomes. **a** Dot plots overlaid onto boxplots showing expression levels of biomarker candidates that are consistently detected in at least two independent MS-based AD CSF proteome studies in the cortex and CSF proteomes. The *p* values of the DE analyses between AD and healthy control are displayed on the top of the plots. DE analyses were carried out through the LIMMA R package. X-axis shows sample groups, y-axis indicates Log2 transformed TMT intensity. Boxplot center line, median; box limits, upper and lower quartiles; whiskers, 1.5x interquartile range; points, expression levels of each individual samples. **b** Dot plots overlaid onto boxplots showing expression levels of novel biomarker candidates detected in our ultra-deep proteome in cortex and CSF datasets. The *p* values of the DE analyses between AD and healthy control are displayed on the top of the plots. DE analyses were carried out through the LIMMA R package. X-axis shows sample groups, y-axis indicates Log2 transformed TMT intensity. Boxplot center line, median; box limits, upper and lower quartiles; whiskers, 1.5x interquartile range; points, expression levels of each individual samples

To evaluate the reliability and reproducibility of these 44 DE proteins across laboratories, we compared our CSF proteome with two independent MS-based CSF proteomic studies [33, 34]. SMOC1 and C1QTNF5 showed up in all three independent studies. OLFML3, SPON1, and SLIT2 stood out in this study and reference study 1 (data vi) [33]. GPNMB emerged in this study and reference study 2 (data viii) [34] (Fig. 3c, d). All six proteins were reported to be tightly associated with AD pathogenesis [18]. While, SMOC1 and GPNMB have been reported as putative CSF biomarkers in previous studies [18, 33, 62], C1QTNF5, OLFML3, SPON1 and SLIT2 are novel candidates that show reproducibility across distinct laboratories and pipelines (e.g. depleted vs undepleted CSF). Notably, the expression level of all six proteins started to raise in the cortex of mild cognitive impairment patients, implicating their potential as early diagnosis biomarkers for Alzheimer’s disease (Fig. 4a). Moreover, our CSF proteome profiling also discovered potential AD biomarkers of low abundance that were beyond the detection limits of other studies. For instance, the abundance rankings of SLC5A3, BBOX1, CAMK4, and CAMKK2 in the CSF were 3039, 3040, 4191, and 5787, respectively, all beyond the detecting limits of previously reported studies (Figs. 3d, 4b). The levels of CAMK4 and CAMKK2 were decreased in both cortex and CSF. Finally, HTRA1, a possible genetic risk factor for AD and an enzyme that degrades ApoE4 and APP [63, 64], was also revealed as a novel DE protein in our CSF proteome (Fig. 4b). Collectively, the integration of CSF and cortex proteomes unveils consistent CSF biomarker candidates in AD.

### Integration of human and mouse CSF proteomes identifies consistent mitochondrial protein decrease in Alzheimer’s disease

**Fig. 5:**
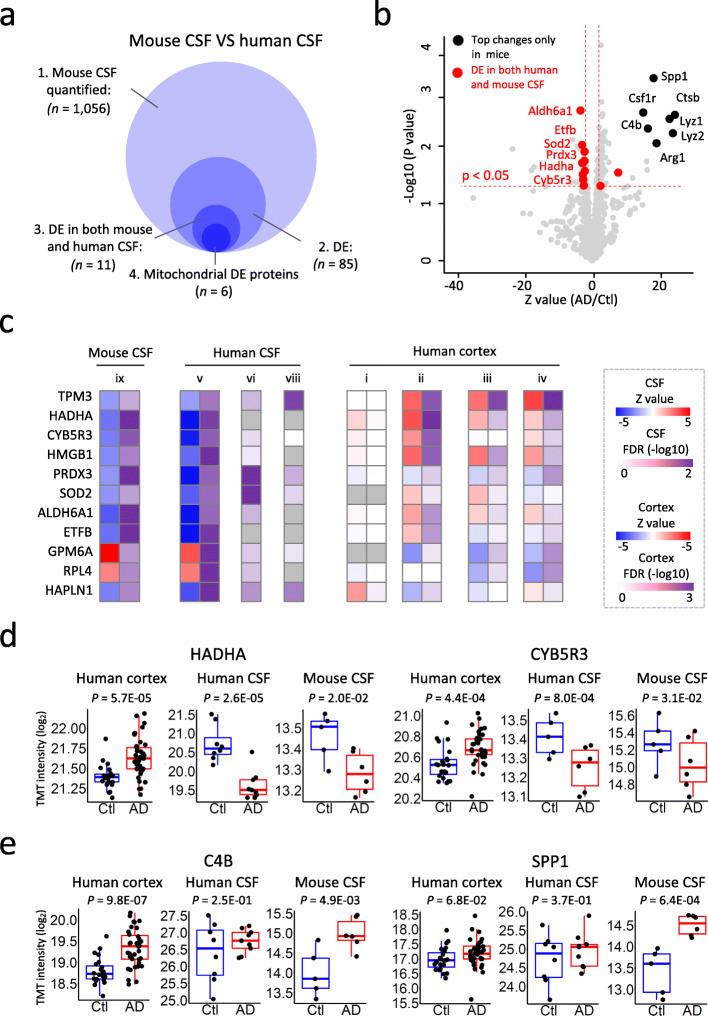
Integration of human and mouse CSF proteomes identifies consistent mitochondrial protein decrease in Alzheimer’s disease. **a** Summary of the DE analysis of mouse CSF proteome, and its integration with human CSF proteome. The analysis was carried out in 4 steps. 1) 1056 proteins were quantified in 6 5XFAD and 5 WT groups that were pooled from 32 mice. 2) 85 proteins were identified as DE proteins with a Z score difference > 2 and *p* value < 0.05. 3) 11 out of these 85 proteins are differentially expressed in both human and mouse CSF samples. 4) 6 out of these 11 DE proteins are mitochondrial proteins. **b** Volcano plot for quantified mouse CSF proteome. X-axis shows the Z score transformed log2 fold changes comparing AD to Ctl. Y-axis shows the -log10 *p* value. Top DE proteins in mice CSF are plotted in black and labeled. Proteins that are differentially expressed in both human and mouse CSF are plotted in red, and mitochondrial proteins are further labeled. Red dashed lines indicate the DE cutoff of *p* value < 0.05 and Z score difference > 2. **c** Heatmap shows Z score, −log10 FDR value or *p* value of the 11 DE proteins in mouse CSF, human CSF, and human cortex proteomes. **d** Expression levels of representative Mitochondrial DE proteins in human cortex, human CSF and mouse CSF. Dot plots overlaid onto boxplots showing expression of representative DE proteins in panel c and d. The *p* values of the DE analyses between AD and healthy control are displayed on the top of the plots. DE analyses were carried out through the LIMMA R package. X-axis shows sample groups, y-axis indicates Log2 transformed TMT intensity. Boxplot center line, median; box limits, upper and lower quartiles; whiskers, 1.5x interquartile range; points, expression levels of each individual samples. **e** Expression levels of representative top DE proteins in human cortex and mouse CSF. Dot plots overlaid onto boxplots showing expression of representative DE proteins in panel c and d. The *p* values of the DE analyses between AD and healthy control are displayed on the top of the plots. DE analyses were carried out through the LIMMA R package. X-axis shows sample groups, y-axis indicates Log2 transformed TMT intensity. Boxplot center line, median; box limits, upper and lower quartiles; whiskers, 1.5x interquartile range; points, expression levels of each individual samples

CSF biomarkers that are conserved in human and mouse models are valuable for the AD community to explore AD-related molecular mechanism. Here we conducted proteomic analysis to identify Aβ-induced protein changes in CSF from 5xFAD mouse, in which mutant APP and PSEN1 are overexpressed to generate a high level of Aβ peptide. An 11-plex TMT-LC/LC-MS/MS analysis (6 samples from 5xFAD and 5 samples from age-matched wild type mice) allowed the quantification of 1056 mouse CSF proteins, with 85 DE proteins (Z value > 2 and *p* value < 0.05, Fig. 5a, b). Eleven out of these 85 proteins were overlaid with the human CSF DE proteins (Fig. 5c). Strikingly more than 50% of these consistent DEs are from mitochondria, suggesting that mitochondrial dysfunction is highly conserved in AD and the 5xFAD mouse. Many of these mitochondrial proteins were changed in AD cortex with an expression pattern similar to Aβ42 peptide (i.e. increase in cortex and decrease in CSF), such as HADHA and CYB5R3 (Fig. 5c, d). We also discovered C4B and SPP1 that are known to be tightly associated with AD pathogenesis among the top DE proteins in the mouse CSF (Fig. 5b). We detected the increase of C4B and SPP1 in AD cortex but failed to detect their significant changes in our small human CSF cohort (Fig. 5e). In summary, the integrative analysis of mouse and human CSF elucidated Aβ-induced protein changes in mouse CSF and unveiled consistent mitochondrial disorder in AD in both human and mouse CSF.

### Integration of CSF, serum, and cortex proteomes indicates consistent mitochondrial signatures in Alzheimer’s disease

**Fig. 6:**
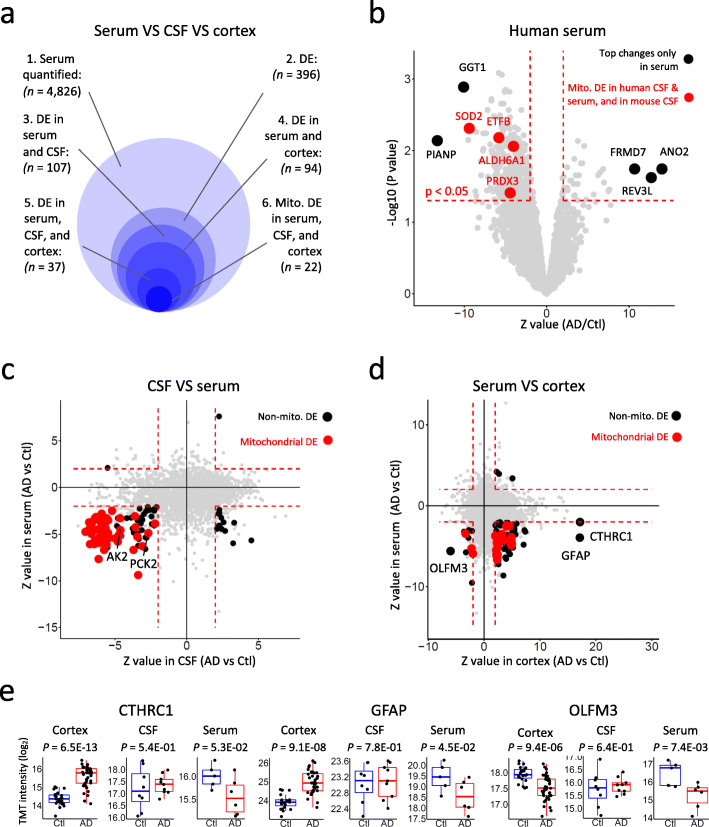
Integration of CSF, serum, and cortex datasets elucidates consistent mitochondrial signatures in Alzheimer’s disease across proteomes. **a** Summary of the DE analysis of serum proteome, and its integration with CSF and cortex proteome. Four thousand eight hundred twenty-six proteins were quantified in AD serum samples. Three hundred ninety-five proteins were identified as differentially expressed proteins with a Z-score transformed log2 fold difference > 2 and *p* value < 0.05. One hundred seven out of these proteins are differentially expressed in both serum and CSF proteomes, 94 of these proteins are differentially expressed in both serum and brain proteomes, 37 proteins are differentially expressed in serum, CSF, and Cortex proteome. Twenty-two out of the 37 DE proteins are mitochondrial proteins. **b** Deep serum proteome analysis identifies decrease of mitochondrial proteins. The X-axis of the volcano plot shows the Z score transformed log2 fold change comparing AD to Ctl and Y-axis indicates -log10 *p* value. Top DE proteins in serum are plotted in black and labeled. Proteins that are differentially expressed in human CSF, mouse CSF, and human serum proteomes are plotted in red and labeled. Red dashed lines indicate the DE cutoff of *p* value < 0.05 and Z value difference > 2. **c** Integration of serum and CSF proteomes identifies consistent and massive mitochondrial protein decrease. Proteins that are quantified in both serum and CSF are plotted as a function of their Z values comparing AD to Ctl in CSF (x-axis) and in serum (y-axis). Fifty-five non-mitochondrial DE proteins with Z value difference > 2 and FDR < 0.2 in CSF or *p* value < 0.05 in serum are plotted in black, and 52 mitochondrial DE proteins are plotted in red. Names of two mitochondrial proteins that were applied for TOMAHAQ targeted MS assay (Fig. 8) were labeled. Red dashed lines indicate Z value difference > 2 in CSF and serum. **d** Integration of serum and cortex proteomes unveils mitochondrial protein changes and amyloid-correlated protein panel. Proteins that are quantified in both serum and cortex are plotted as a function of their Z value change comparing AD to Ctl in cortex (x-axis) and in serum (y-axis). Seventy non-mitochondrial DE proteins with Z value difference > 2 and FDR < 0.2 in cortex or *p* value < 0.05 in serum were plotted in black. Three top DE proteins that were reported in the amyloid-correlated protein panel in previous cortex study were labeled.Twenty-four mitochondrial DE proteins are plotted in red. Red dashed lines indicate Z value difference > 2 in serum and cortex. **e** Expression levels of representative DE proteins in cortex and serum. Dot plots overlaid onto boxplots showing expression of representative DE proteins in cortex, CSF, and serum proteomes. The *p* values of the DE analyses between AD and healthy control are displayed on the top of the plots. DE analyses were carried out through the LIMMA R package. X-axis shows sample groups, y-axis indicates Log2 transformed TMT intensity. Boxplot center line, median; box limits, upper and lower quartiles; whiskers, 1.5x interquartile range; points, expression levels of each individual samples

Compared with CSF biomarkers, blood-based biomarkers are more promising for first-line diagnosis and are urgently needed. We systematically compared the CSF, serum and cortex proteomes to investigate AD pathogenesis signatures. An ultra-deep serum profiling of 6 AD and 5 healthy control cases was performed to quantify 4826 unique proteins [25]. As the serum samples are often contaminated by proteins from red blood cells, we first corrected this variable by a linear regression model-based approach [49], and then defined 396 DE proteins (Z value > 2 and *p* value < 0.05). Comparison with DE proteins in CSF and cortex led to 94 DE proteins in serum and cortex, 107 DE proteins in serum and CSF, and 37 proteins in all three layers of proteomes. Strikingly, 22 out of these 37 proteins are mitochondrial proteins (Fig. 6a), highlighting mitochondrial changes as the most consistent AD signature across cortex, CSF and serum.

The DE analysis in serum identified several AD relevant changes among the top DE proteins (e.g. GGT1 and ANO2) (Fig. 6b). For example, a study has reported a linear association between serum GGT concentration and the risk of AD [65]. Interestingly, 4 out of the 6 mitochondrial proteins that decreased in AD in both human and mouse CSFs were also reduced in the AD serum (i.e. ALDH6A1, ETFB, SOD2, and PRDX3), highlighting their robustness as the AD biofluid signature (Fig. 6b, Fig. S4). We next investigated proteins that were differentially expressed in both CSF and serum. Fifty-two of total 107 DE proteins were mitochondrial proteins, showing decreased levels in AD in both serum and CSF (Fig. 6c). We further examined the total 94 DE proteins in serum and cortex and found that most of these proteins were increased in cortex and decreased in serum, including 21 mitochondrial proteins (Fig. 6d), which is reminiscent of the distribution pattern of Aβ peptides (higher in cortex and lower in serum in AD cases) [66]. The accumulation of proteins in the cortex may be resulted, at least partially, from prominent protein aggregation in the brain. Indeed, we previously identified the deposition of mitochondrial components in the profiling of aggregated proteome in AD brain [67]. Interestingly AD-correlated protein panel of CTHRC1, GFAP and OLFM3 in brain [18] were revealed as top DE proteins in AD serum (Fig. 6d, e). Together, the integrated analysis shows mitochondrial protein changes as the most consistent AD signature carried over from brain cortex to CSF and serum.

### Integrating the rankings of ten individual datasets through order statistics prioritizes top AD protein signatures

**Fig. 7:**
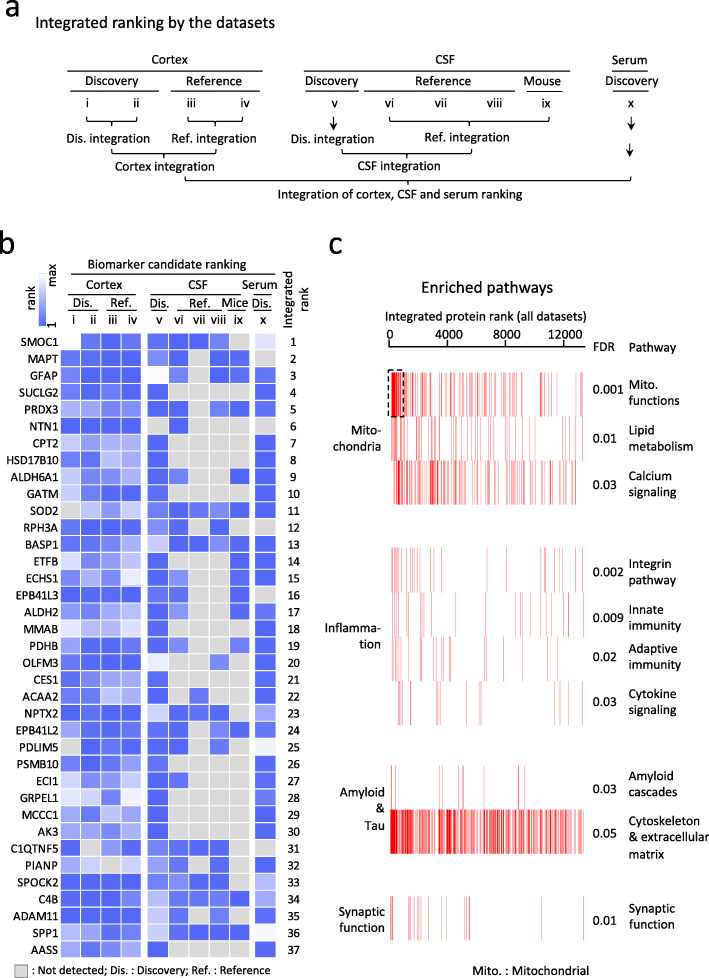
Integrating the protein rankings in individual datasets though order statistics prioritizes top AD signatures. **a** Workflow for tiered integration of individual proteome ranking by order statistics. Rank of each individual dataset was integrated by discovery or reference cohorts separately first, and was then combined into cortex, CSF, or serum ranking. Lastly, the three ranks were integrated into a final integrative ranking. **b** Top protein signatures of Alzheimer’s disease prioritized through the integrated ranking. Heatmap shows the ranking of top AD signature proteins with a final integrated ranking *p* value < 0.001 in each of the ten datasets. Protein ranks are labeled on the right side of the heatmap. The rankings of proteins are shown by boxes of two-color gradients, with missing values indicated by grey boxes. **c** Mitochondrial function is the most significantly enriched pathway in the integrated ranking. Pathways are enriched by GSEA and further categorized into four groups. The barcode plots represent the positions of proteins in the sorted integrated ranking

**Fig. 8:**
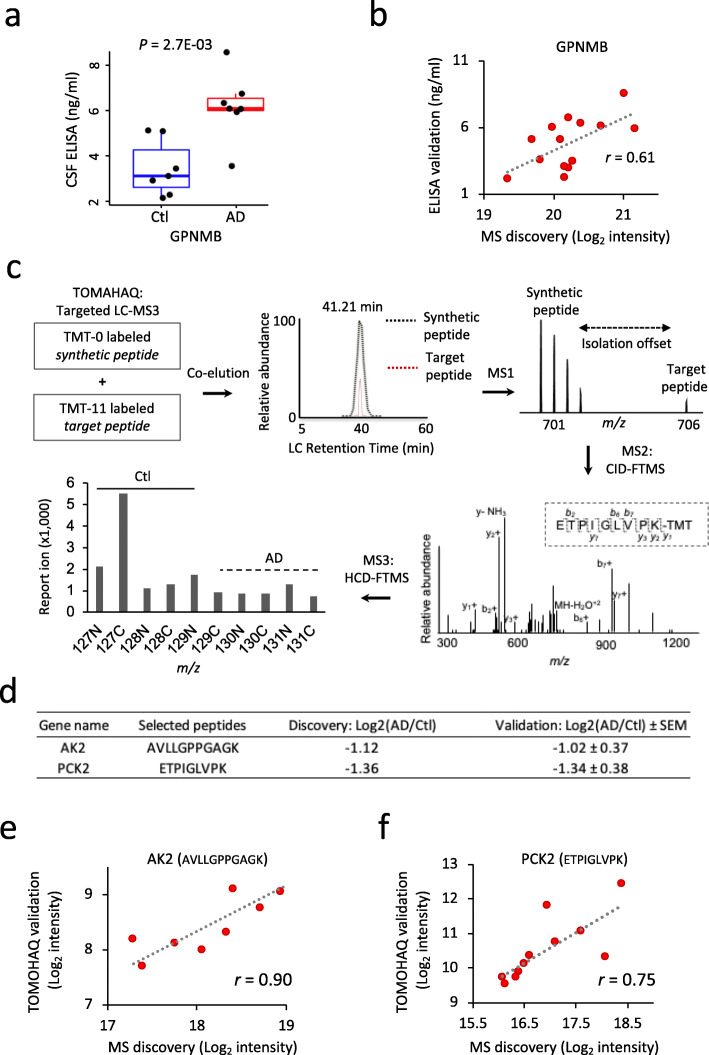
Validation of MS discoveries by ELISA and TOMAHAQ assays **a**) Validation of MS discoveries by ELISA assay. Dots overlaid onto boxplots showing expression level of the AD biomarker candidate GPNMB in CSF quantified by ELISA assay. Seven healthy control and 7 AD samples were analyzed. The *p* value of the DE analysis between AD and healthy control by Student’s t-test is displayed on the top of the plots. X-axis shows sample groups, y-axis indicates the ELISA measurement of GPNMB concentration in CSF (ng/ml). Boxplot center line, median; box limits, upper and lower quartiles; whiskers, 1.5x interquartile range; points, expression levels of individual samples. **b** Scatter plot shows the correlation between the ELISA and the discovery MS data. Pearson correlation coefficient (*r*) is displayed. X-axis shows the Log_2_TMT ion intensities of GPNMB quantified by MS. Y-axis indicates the CSF concentration of GPNMB (ng/ml) quantified by ELISA. **c** Workflow for the TOMAHAQ targeted MS validation assay. A synthetic trigger peptide was spiked into the mixture of multiplexed samples to validate the quantification of a candidate biomarker peptide. The synthetic peptide and native peptide were co-eluted, and the synthetic peptide was presented at high concentration, triggering the MS instrument to quantify the native peptide by MS3 using a predefined isolation offset. MS3 ions were produced by pre-determined y or b ions from targeted MS2 spectra, and the resulting reporter ions were applied for the quantification of the targeted biomarker candidate. **d** Comparison of the TOMAHAQ results and the discovery MS data. **e** Scatter plot shows the correlation between the TOMAHAQ and the discovery MS results of AK2 in the human CSF samples. Pearson correlation coefficient (*r*) is displayed. X- and Y- axes indicate the Log_2_ TMT intensities from the discovery MS and TOMAHAQ assay, respectively. **f** Scatter plot shows the correlation between the TOMAHAQ and the discovery MS results of PCK2 in the human CSF samples

Integration of multiple dimensions of data has proven powerful for prioritizing core disease proteins and pathways [17, 18, 49]. Here we extended this idea by combining datasets from distinct AD tissue/biofluids and independent studies to rank disease proteins and pathways using order statistics [53] and gene set enrichment analysis (GSEA). The integration was carried out in a 3-step manner. Specifically, discovery cohorts or reference cohorts were separately combined. Proteomes of individual tissue/biofluids were then combined into cortex, CSF, or serum datasets for ranking. Finally, the three ranks were integrated into a final rank (Fig. 7a, Supplemental Table S7). SMOC1 and tau proteins were ranked the top 2 of the list, consistent with many previous AD biomarker studies. Other proteins such as GFAP, NTN1, OLFM3, NPTX2, C1QTNF5, C4B, and SPP1 were also showed up as top proteins, agreeing with our current understanding of AD pathogenesis. Moreover, mitochondrial proteins were ranked high in the list as well (e.g. SUCLG2, PRDX3, CPT2, HSD17B10, ALDH6A1, GATM, and SOD2) (Fig. 7b). We prioritized signaling pathways by GSEA and identified 10 major pathways (FDR < 0.05; Fig. 7c) out of the 16 core pathways detected in the deep AD cortex study [18]. Collectively these 10 pathways can be classified into 4 major categories including mitochondrial functions, inflammation, amyloid and tau pathway, and synaptic function. Finally, we performed two alternative validation assays to confirm the MS discoveries. ELISA assay was used to analyze the CSF samples of 7 healthy controls and 7 AD cases, confirming the increase of the candidate biomarker GPNMB in the AD samples (Fig. 8a, b). Due to the limitation of available ELISA kits, we also implemented the TOMAHAQ-based targeted MS assay to validate the change of two mitochondrial proteins (AK2 and PCK2) in the CSF samples. In this targeted MS assay, tryptic peptides in AK2 and PCK2 were synthesized as internal standards to guide the quantification of native corresponding peptides (Fig. 8c) [56]. Consistently, both mitochondrial proteins were confirmed to be reduced in the CSF AD samples (Fig. 8d-f). Together, we prioritized a list of promising AD signatures through a systems biology approach. These rich data resources will serve as a foundation for future large-scale biomarker validation studies for the AD community.

## Discussion

Recent breakthroughs, especially in-depth and large-scale omics studies of brain tissue, have dramatically extended our discovery of molecular pathogenesis in AD [18, 49]. New dysregulated genes/proteins/pathways have been increasingly identified and linked to AD pathogenesis, suggesting of multifactorial pathologies in AD. However, in-depth proteome discovery in the proximal body fluids (e.g. CSF and serum/plasma) is still rare, largely due to technical challenges to address the complexity of CSF and serum/plasma proteomes. The relationship between diverse brain pathologies and protein alterations in body fluids is not fully explored at the proteome level. Furthermore, the variable results obtained from studies evaluating proteins involving in amyloid and tau pathology as disease biomarkers underlined the importance of novel biofluid biomarkers [68]. To meet the challenges to analyze CSF and serum/plasma proteomes in AD, we have recently developed an in-depth biofluid profiling platform [25] that combines un-depleted biofluid sample processing, multiplexed TMT labeling, extensive two-dimensional LC fractionation and high-resolution tandem mass spectrometry. The platform enables the quantification of 5941 and 4826 proteins in CSF and serum, respectively, providing the most in-depth biofluid proteome landscape so far for the AD community.

In addition to the issue of proteome coverage, reproducibility is often another concern in many of previously published AD biomarker studies. Even some novel CSF protein biomarker candidates have been proposed, many of them, however, are not successfully repeated across different laboratories, distinct proteomic platform, and/or independent cohorts, raising a substantial bottleneck for selecting reliable candidates for large-scale validation. To address this issue, we systematically integrated our ultra-deep CSF proteome with two other discovery-driven deep CSF proteomic studies in AD, resulting in 6 biomarker candidates that were repeatedly emerged in at least two independent studies including SMOC1, C1QTNF5, OLFML3, SPON1, SLIT2 and GPNMB. Remarkably, all of them were reported to be highly linked to AD pathogenesis in brain tissue [18]. SMOC1 has been shown to accumulate in plaque structures of AD in brain. The expression levels of SMOC1, OLFML3, SLIT2, and GPNMB were highly correlated with the Aβ level in AD brain, and these findings were also recapitulated in the 5xFAD mouse model [18]. Consistently, SMOC1 and GPNMB were reported to be CSF biomarker candidates for AD in other recent biomarker studies [62, 69]. These proteins represent the most promising CSF biomarker candidates of AD for future large-scale studies.

Mitochondrial function and energy metabolism are known to be severely compromised processes repeatedly reported in AD [18, 70]. Emerging lines of evidence suggest the growing importance of mitochondria damage and energy defects in AD pathogenesis [70], and mitochondrial deficit is proposed as a major hallmark of AD pathogenesis besides amyloid and tau pathologies [70]. Studies in a *C. elegans* model expressing pan-neuronal human Aβ show that metabolic stress is a primary pathogenic event [71] and impaired mitochondrial calcium efflux contributes to disease progression [72]. Enhanced mitochondrial proteostasis may reduce amyloid-β proteotoxicity [73] and NAD+ supplementation normalizes key Alzheimer’s features and DNA damage responses in an AD mouse model [74]. Mitochondria have gradually been recognized as a major novel therapeutic target in AD [70]. In this study, we identified consistent and evident mitochondrial protein decreases in AD CSF and serum samples, which have rarely reported until the availability of the deep CSF/serum profiling (our reference datasets) [25, 33]. This is understandable because, as our analysis suggested, high proteome coverage is a prerequisite to detect mitochondrial changes due to their low abundance, explaining why they are missing in numerous previous biofluid studies of shallow proteome coverage. Although the causative factors of mitochondrial dysfunction in AD are not fully understood, we believe that the mitochondrial changes in the cortex, CSF and serum are highly associated in AD based on several lines of evidence. Mitochondrial changes can co-occur with amyloid deposition early in the brain of asymptomatic cases with amyloid pathology, as well as mild cognitive impairment subjects [18, 33, 75]. Amyloid peptides have been reported to directly aggregate in mitochondrial compartment [75, 76]. Recently, vascular deposits of Aβ peptides (amyloid angiopathy) are increasingly recognized as a common pathology in AD cases, supporting that Aβ peptides circulate within the interstitial fluid, including CSF, and blood vessels through perivascular (e.g. lymphatic) drainage pathways during the crosstalk between the brain and the vascular system [77]. As Aβ can form vascular deposition, it is likely that Aβ could lead to mitochondrial damage in the vascular system. This local Aβ-induced mitochondrial damage may partially address an important question – where is the origin of the identified mitochondrial proteins? Interestingly, emerging data suggest that mitochondria can be released into extracellular space, and transferred between cells [78], although mitochondria have traditionally been known as the intracellular powerhouse. For instance, neurons can transfer damaged mitochondria to astrocytes for disposal and recycling, and astrocytes can also release mitochondria to neurons under stress [79]. Astrocytic mitochondria may also be released to the CSF as a biomarker for evaluating brain integrity, with low CSF mitochondrial quantity and activity indicating brain damage [80]. Nevertheless, the origin of the mitochondria proteins is worth future investigation. We acknowledge that our results only indicate a correlation between mitochondria changes in proximal body fluids and brain lesions in AD. Further studies are clearly required to understand the mechanism behinds the associated mitochondrial protein changes in the cortex, CSF and serum in AD.

Here we demonstrate that a mitochondrial signature is the most significant and consistent changes detected across human brain cortex, CSF and serum in AD, and it has been recapitulated in the 5XFAD mice as well. It has been mentioned that these mitochondrial changes can only be confidently detected in an ultra-deep proteomic setting. This exciting finding provides a strong rationale not only for the development of disease diagnostic biomarkers but also the implementation of novel prognostic biomarkers for therapeutic strategies targeting mitochondria in AD.

## Conclusions

In summary, we quantified 13,833, 5941, and 4826 proteins from human cortex, CSF and serum respectively through our newly established TMT-LC/LC-MS/MS platform. We showed evident changes of many mitochondria proteins across AD cortex, CSF, and serum. Through a series of integrated analyses of 10 AD tissue and biofluids proteomic datasets from three independent deep proteomic studies, we revealed a number of AD biomarker candidates of high confidence, providing a rich data resource not only for selecting reproducible candidates for large-scale biomarker validation, but also for exploring protein-mediated cortex-CSF-blood communication during disease progression to reveal disease mechanism that may guide the development of novel therapeutic strategies.

## Supplementary information

**Additional file 1: Supplementary Figure S1.** DE mitochondrial proteins are tightly correlated with each other in human CSF. **S2.** Ultra-deep profiling depth is a prerequisite to detect evident mitochondrial signatures presented in AD CSF. **S3.** PPI module proteins are highly correlated with each other. **S4.** Mitochondrial proteins that have decreased expression levels in the examined biofluids in both human and mouse AD.

**Additional file 2: Supplementary Table S1.** Differential expression analysis of whole proteome from Human brain cortex tissues (data i). **S2**. Differential expression analysis of whole proteome from Human brain cortex tissues (data ii). **S3.** Differential expression analysis of whole proteome profiling of Human CSF (data v). **S4.** Differential expression analysis of whole proteome profiling of Mouse CSF (data ix). **S5.** Differential expression analysis of whole proteome from human serum (data x). **S6.** Previously reported AD CSF biomarker candidates. **S7.** Integrated ranking of proteins in all ten datasets

## Data Availability

The proteomics data used in this study are available via the AD Knowledge Portal (https://adknowledgeportal.synapse.org). The Banner Brain and Body Donation Program cortex, CSF and serum TMT proteomics data are available through 10.7303/syn21638690. The Mount Sinai Brain Bank cortex TMT proteomics data are accessible through 10.7303/syn21347564, and additional information can be found at 10.7303/syn7392158. The mouse CSF TMT proteomics data are accessible through Proteome Xchange Consortium (http://www.proteomexchange.org) via the PRIDE partner repository with the dataset identifiers PXD018658.

## Abbreviations

AD: Alzheimer’s disease
MS: Mass spectrometry
TMT: Tandem mass tags
LC/LC-MS/MS: Two-dimensional liquid chromatography coupled with tandem mass spectrometry

## References

[1] Alzheimer’s A (2016). 2016 Alzheimer’s disease facts and figures. Alzheimers Dement.

[2] Braak H, Braak E (1995). Staging of Alzheimer’s disease-related neurofibrillary changes. Neurobiol Aging.

[3] Hardy J, Selkoe DJ (2002). The amyloid hypothesis of Alzheimer’s disease: progress and problems on the road to therapeutics. Science.

[4] Selkoe DJ, Hardy J (2016). The amyloid hypothesis of Alzheimer’s disease at 25 years. EMBO Mol Med.

[5] McKhann GM (2011). The diagnosis of dementia due to Alzheimer’s disease: recommendations from the National Institute on Aging-Alzheimer’s Association workgroups on diagnostic guidelines for Alzheimer’s disease. Alzheimers Dement.

[6] Wolk DA, Dickerson BC (2018). Clinical features and diagnosis of Alzheimer disease.

[7] Janelidze S (2020). Plasma P-tau181 in Alzheimer’s disease: relationship to other biomarkers, differential diagnosis, neuropathology and longitudinal progression to Alzheimer’s dementia. Nat Med.

[8] Thijssen EH (2020). Diagnostic value of plasma phosphorylated tau181 in Alzheimer’s disease and frontotemporal lobar degeneration. Nat Med.

[9] Karikari TK, Ashton NJ, Janelidze S, Benedet AL, Rodriguez JL, Chamoun M, Savard M, Kang MS, Therriault J, Schöll M, Massarweh G, Soucy J-P, Höglund K, Gunnar B, Mattsson N, PalmqvisT S, Gauthier S, Kaj B, T.A.P (2020). Blood phosphorylated tau 181 as a biomarker for Alzheimer’s disease: a diagnostic performance and prediction modelling study using data from four prospective cohorts. Lancet Neurol.

[10] Cook D (2014). Lessons learned from the fate of AstraZeneca's drug pipeline: a five-dimensional framework. Nat Rev Drug Discov.

[11] Hampel H (2018). Blood-based biomarkers for Alzheimer disease: mapping the road to the clinic. Nat Rev Neurol.

[12] Sperling RA (2011). Toward defining the preclinical stages of Alzheimer’s disease: recommendations from the National Institute on Aging-Alzheimer’s Association workgroups on diagnostic guidelines for Alzheimer’s disease. Alzheimers Dement.

[13] Blennow K (2017). A review of fluid biomarkers for Alzheimer’s disease: moving from CSF to blood. Neurol Ther.

[14] Zhang Y (2013). Protein analysis by shotgun/bottom-up proteomics. Chem Rev.

[15] Aebersold R, Mann M (2016). Mass-spectrometric exploration of proteome structure and function. Nature.

[16] Mertins P (2016). Proteogenomics connects somatic mutations to signalling in breast cancer. Nature.

[17] Stewart E (2018). Identification of therapeutic targets in rhabdomyosarcoma through integrated genomic, epigenomic, and proteomic analyses. Cancer Cell.

[18] Bai B (2020). Deep multilayer brain proteomics identifies molecular networks in Alzheimer’s disease progression. Neuron.

[19] Altelaar AF, Munoz J, Heck AJ (2013). Next-generation proteomics: towards an integrative view of proteome dynamics. Nat Rev Genet.

[20] Ludwig C (2018). Data-independent acquisition-based SWATH-MS for quantitative proteomics: a tutorial. Mol Syst Biol.

[21] Rauniyar N, Yates JR (2014). Isobaric labeling-based relative quantification in shotgun proteomics. J Proteome Res.

[22] Thompson A (2019). TMTpro: design, synthesis, and initial evaluation of a proline-based isobaric 16-Plex tandem mass tag reagent set. Anal Chem.

[23] Ting L (2011). MS3 eliminates ratio distortion in isobaric multiplexed quantitative proteomics. Nat Methods.

[24] Niu M (2017). Extensive peptide fractionation and y1 ion-based interference detection method for enabling accurate quantification by isobaric labeling and mass spectrometry. Anal Chem.

[25] Dey KK (2019). Deep undepleted human serum proteome profiling toward biomarker discovery for Alzheimer’s disease. Clin Proteomics.

[26] Wang H (2019). Deep multiomics profiling of brain tumors identifies signaling networks downstream of cancer driver genes. Nat Commun.

[27] Anderson NL, Anderson NG (2002). The human plasma proteome: history, character, and diagnostic prospects. Mol Cell Proteomics.

[28] Arican O (2005). Serum levels of TNF-alpha, IFN-gamma, IL-6, IL-8, IL-12, IL-17, and IL-18 in patients with active psoriasis and correlation with disease severity. Mediat Inflamm.

[29] Geyer PE (2017). Revisiting biomarker discovery by plasma proteomics. Mol Syst Biol.

[30] Pieper R (2003). Multi-component immunoaffinity subtraction chromatography: an innovative step towards a comprehensive survey of the human plasma proteome. Proteomics.

[31] Qian WJ (2008). Enhanced detection of low abundance human plasma proteins using a tandem IgY12-SuperMix immunoaffinity separation strategy. Mol Cell Proteomics.

[32] Tu C (2010). Depletion of abundant plasma proteins and limitations of plasma proteomics. J Proteome Res.

[33] Higginbotham L, et al. Integrated proteomics reveals brain-based cerebrospinal fluid biomarkers in asymptomatic and symptomatic Alzheimer’s disease. bioRxiv. 2019. 10.1101/806752. 10.1126/sciadv.aaz9360 PMC7577712 33087358

[34] Sathe G (2019). Quantitative proteomic profiling of cerebrospinal fluid to identify candidate biomarkers for Alzheimer’s disease. Proteomics Clin Appl.

[35] Beach TG (2015). Arizona study of aging and neurodegenerative disorders and brain and body donation program. Neuropathology.

[36] Wang M (2018). The Mount Sinai cohort of large-scale genomic, transcriptomic and proteomic data in Alzheimer’s disease. Sci Data.

[37] Liu L, Duff K (2008). A technique for serial collection of cerebrospinal fluid from the cisterna magna in mouse. J Vis Exp.

[38] Bai B (2017). Deep profiling of proteome and phosphoproteome by isobaric labeling, extensive liquid chromatography, and mass spectrometry. Methods Enzymol.

[39] Xu P, Duong DM, Peng JM (2009). Systematical optimization of reverse-phase chromatography for shotgun proteomics. J Proteome Res.

[40] Pagala VR (2015). Quantitative protein analysis by mass spectrometry. Methods Mol Biol.

[41] Wang H (2015). Systematic optimization of long gradient chromatography mass spectrometry for deep analysis of brain proteome. J Proteome Res.

[42] Cheng Y (2018). Partial loss of psychiatric risk gene Mir137 in mice causes repetitive behavior and impairs sociability and learning via increased Pde10a. Nat Neurosci.

[43] Wang X (2014). JUMP: a tag-based database search tool for peptide identification with high sensitivity and accuracy. Mol Cell Proteomics.

[44] Li Y (2016). JUMPg: an integrative Proteogenomics pipeline identifying unannotated proteins in human brain and cancer cells. J Proteome Res.

[45] Shi H (2019). Amino acids license kinase mTORC1 activity and treg cell function via small G proteins rag and rheb. Immunity.

[46] Peng J (2003). Evaluation of multidimensional chromatography coupled with tandem mass spectrometry (LC/LC-MS/MS) for large-scale protein analysis: the yeast proteome. J Proteome Res.

[47] Nesvizhskii AI, Aebersold R (2005). Interpretation of shotgun proteomic data: the protein inference problem. Mol Cell Proteomics.

[48] Geyer PE (2019). Plasma proteome profiling to detect and avoid sample-related biases in biomarker studies. EMBO Mol Med.

[49] Zhang B (2013). Integrated systems approach identifies genetic nodes and networks in late-onset Alzheimer’s disease. Cell.

[50] Ritchie ME (2015). Limma powers differential expression analyses for RNA-sequencing and microarray studies. Nucleic Acids Res.

[51] Benjamini Y, Hochberg Y (1995). Controlling the false discovery rate: a practical and powerful approach to multiple testing. J Roy Statist Soc B.

[52] Ihaka R, Gentleman R (1996). R: a language for data analysis and graphics. J Comput Graph Stat.

[53] Aerts S (2006). Gene prioritization through genomic data fusion. Nat Biotechnol.

[54] Zhang J (2012). A novel retinoblastoma therapy from genomic and epigenetic analyses. Nature.

[55] Subramanian A (2005). Gene set enrichment analysis: a knowledge-based approach for interpreting genome-wide expression profiles. Proc Natl Acad Sci U S A.

[56] Erickson BK (2017). A strategy to combine sample multiplexing with targeted proteomics assays for high-throughput protein signature characterization. Mol Cell.

[57] Uhlen M (2015). Proteomics. Tissue-based map of the human proteome. Science.

[58] Lan J (2018). Systematic evaluation of the use of human plasma and serum for mass-spectrometry-based shotgun proteomics. J Proteome Res.

[59] Ashton NJ (2019). A plasma protein classifier for predicting amyloid burden for preclinical Alzheimer’s disease. Sci Adv.

[60] Hansson O (2019). Advantages and disadvantages of the use of the CSF amyloid beta (Abeta) 42/40 ratio in the diagnosis of Alzheimer’s disease. Alzheimers Res Ther.

[61] Szklarczyk D (2015). STRING v10: protein-protein interaction networks, integrated over the tree of life. Nucleic Acids Res.

[62] Huttenrauch M (2018). Glycoprotein NMB: a novel Alzheimer’s disease associated marker expressed in a subset of activated microglia. Acta Neuropathol Commun.

[63] Grau S (2005). Implications of the serine protease HtrA1 in amyloid precursor protein processing. Proc Natl Acad Sci U S A.

[64] Chu Q (2016). HtrA1 proteolysis of ApoE in vitro is allele selective. J Am Chem Soc.

[65] Kunutsor SK, Laukkanen JA (2016). Gamma glutamyltransferase and risk of future dementia in middle-aged to older Finnish men: a new prospective cohort study. Alzheimers Dement.

[66] Janelidze S (2016). Plasma β-amyloid in Alzheimer’s disease and vascular disease. Sci Rep.

[67] Bai B (2013). U1 small nuclear ribonucleoprotein complex and RNA splicing alterations in Alzheimer’s disease. Proc Natl Acad Sci U S A.

[68] Cummings J (2016). Drug development in Alzheimer’s disease: the path to 2025. Alzheimers Res Ther.

[69] Dayon L (2018). Alzheimer disease pathology and the cerebrospinal fluid proteome. Alzheimers Res Ther.

[70] Cenini G, Voos W (2019). Mitochondria as potential targets in Alzheimer disease therapy: an update. Front Pharmacol.

[71] Teo E (2019). Metabolic stress is a primary pathogenic event in transgenic Caenorhabditis elegans expressing pan-neuronal human amyloid beta. eLife.

[72] Jadiya P (2019). Impaired mitochondrial calcium efflux contributes to disease progression in models of Alzheimer’s disease. Nat Commun.

[73] Sorrentino V (2017). Enhancing mitochondrial proteostasis reduces amyloid-beta proteotoxicity. Nature.

[74] Hou Y (2018). NAD+ supplementation normalizes key Alzheimer’s features and DNA damage responses in a new AD mouse model with introduced DNA repair deficiency. Proc Natl Acad Sci.

[75] Du H (2010). Early deficits in synaptic mitochondria in an Alzheimer’s disease mouse model. Proc Natl Acad Sci U S A.

[76] Anandatheerthavarada HK, Devi L (2007). Amyloid precursor protein and mitochondrial dysfunction in Alzheimer’s disease. Neuroscientist.

[77] Greenberg SM (2020). Cerebral amyloid angiopathy and Alzheimer disease - one peptide, two pathways. Nat Rev Neurol.

[78] Chandel NS (2014). Mitochondria as signaling organelles. BMC Biol.

[79] Hayakawa K (2016). Transfer of mitochondria from astrocytes to neurons after stroke. Nature.

[80] Chou SH (2017). Extracellular mitochondria in cerebrospinal fluid and neurological recovery after subarachnoid hemorrhage. Stroke.

