# Supplementary material for: Integrated analysis of ultra-deep proteomes in cortex, cerebrospinal fluid and serum reveals a mitochondrial signature in Alzheimer’s disease
Source: Mol Neurodegener. 2020 Jul 25;15:43. doi: 10.1186/s13024-020-00384-6 (PMC7382148; doi:10.1186/s13024-020-00384-6)
Supplement: Supplementary file 1 — Additional file 1: Supplementary Figure S1. DE mitochondrial proteins are tightly correlated with each other in human CSF. S2. Ultra-deep profiling depth is a prerequisite to detect evident mitochondrial signatures presented in AD CSF. S3. PPI module proteins are highly correlated with each other. S4. Mitochondrial proteins that have decreased expression levels in the examined biofluids in both human and mouse AD. [file 13024_2020_384_MOESM1_ESM.pdf]

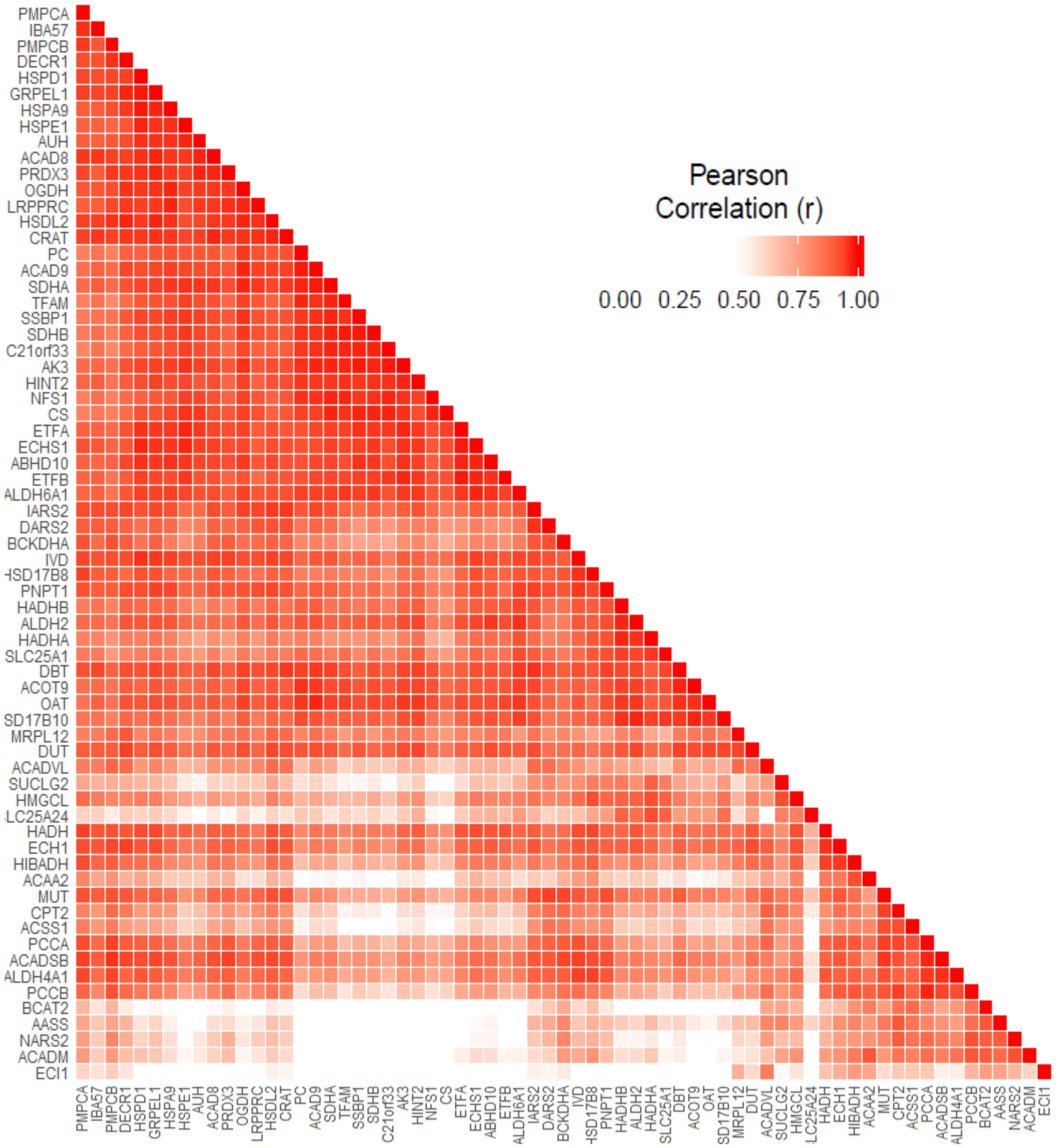

Supplementary Figure 1: **Majority of top differentially expressed (DE) mitochondrial proteins are tightly correlated with each other in the cerebrospinal fluid (CSF)**

Pairwise Pearson correlation analysis was performed for the top DE mitochondrial proteins. Heatmap shows the Pearson correlation coefficient (r) values of top DE mitochondrial proteins.

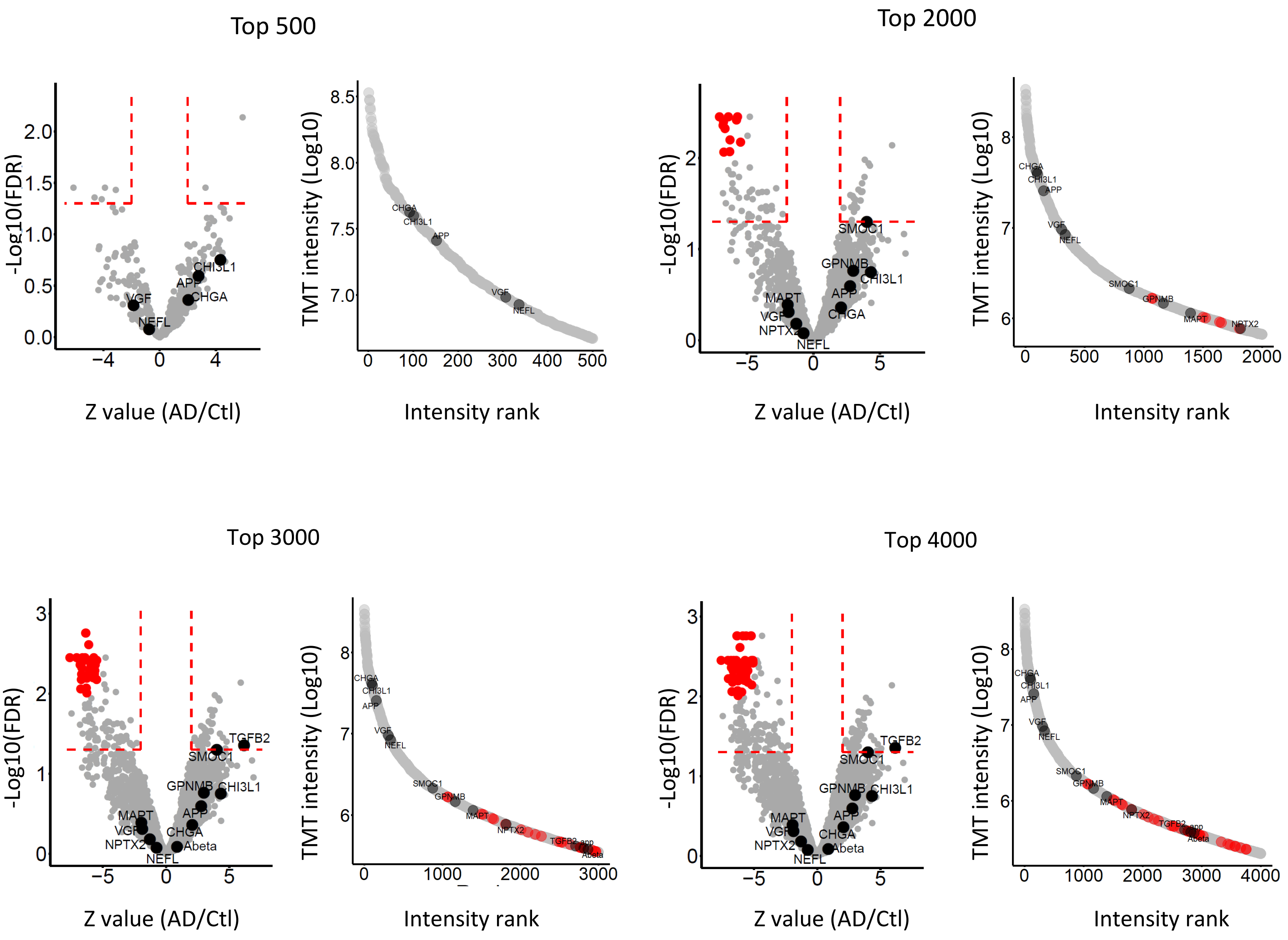

Supplementary Figure 2: **Ultra-deep profiling depth is a prerequisite to detect evident mitochondrial signatures presented in AD CSF proteome**

Volcano plots and corresponding scatter plots of the top 500, 2000, 3000, 4000 most abundant proteins. For the volcano plots, X-axis shows the Z value of the Z score transformed $\log_2$ level fold changes comparing AD to Ctl. Y-axis shows the $-\log_{10}$ level FDR value. Previously reported AD CSF biomarkers are plotted in black and labeled. Top DE mitochondrial proteins with FDR < 0.01 and Z value < -5 are plotted in red. For the scatter plots, CSF proteins are plotted as a function of their concentration rank (*x*-axis) and their mean $\log_{10}$ level TMT intensity in all samples (*y*-axis). Previously reported AD CSF biomarkers are plotted in black. Top DE mitochondrial proteins with Z score difference > 5 and FDR < 0.01 were plotted in red.

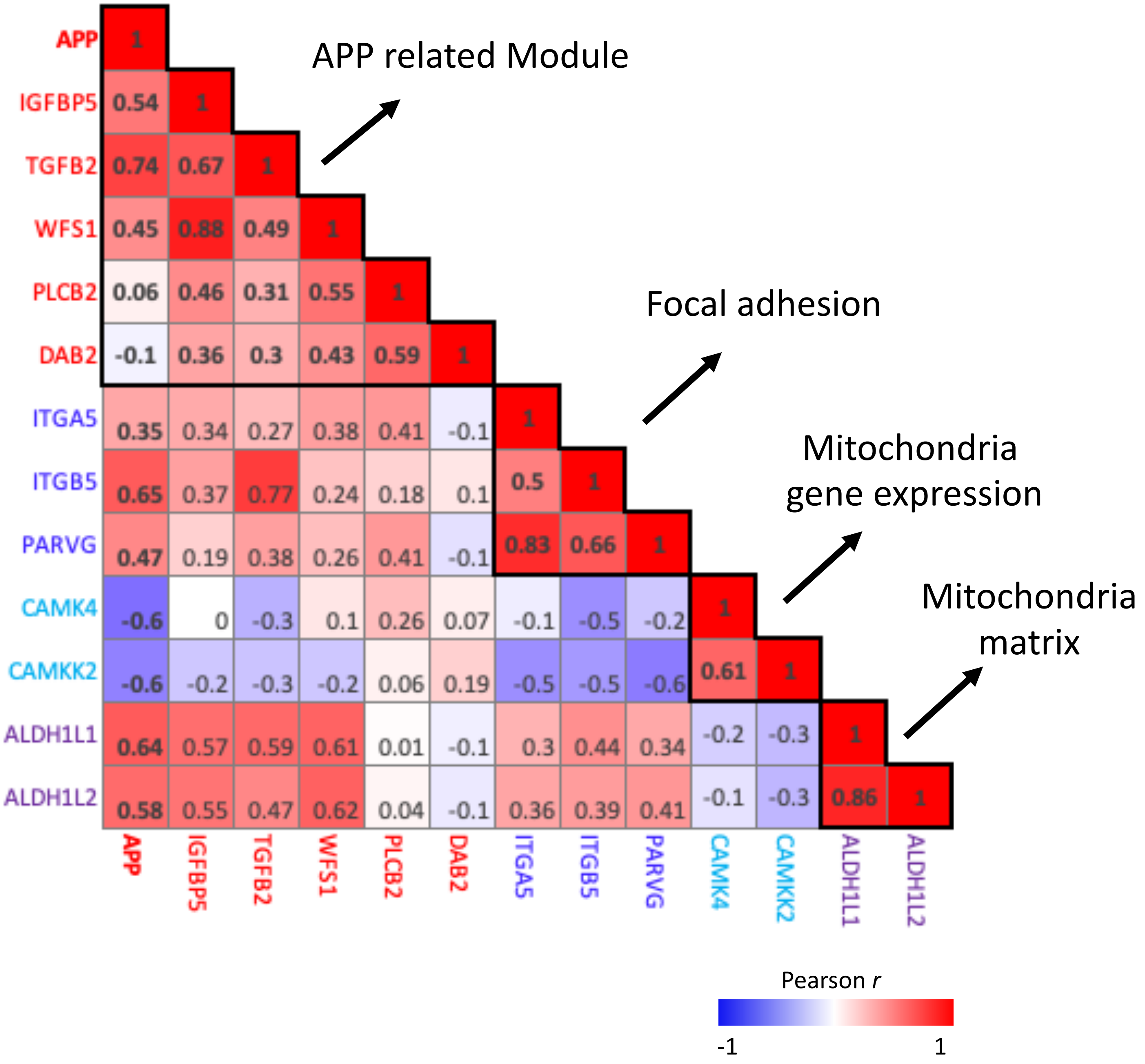

Supplementary Figure 3: **PPI module proteins are highly correlated with each other**

Heatmap shows pairwise correlation analysis of PPI proteins detected in Figure 3E. Pearson correlation coefficient *r* values of each pairwise correlation analysis in the cortex are displayed. The color of each module matches the PPI module edge colors in Figure 3E.

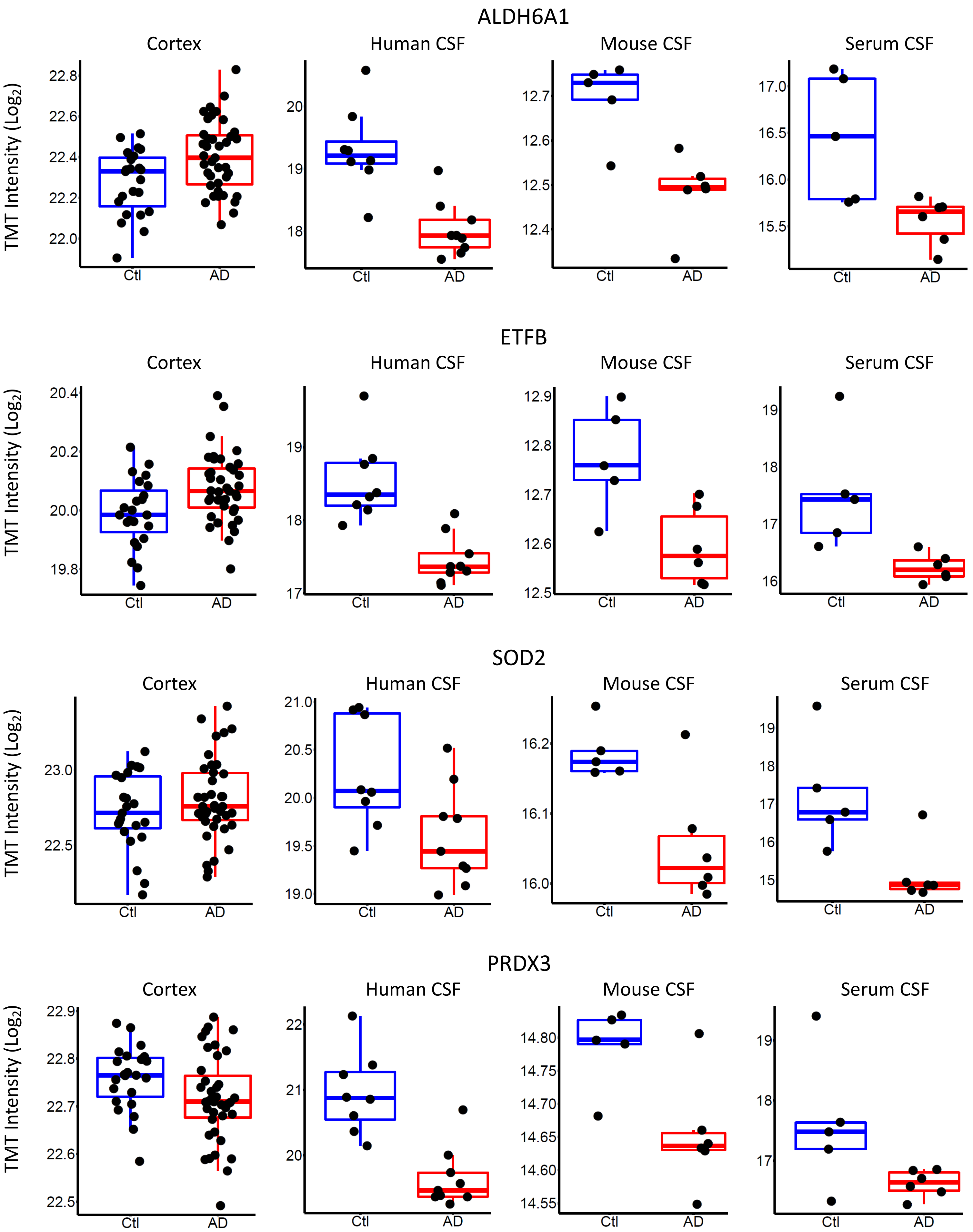
ALDH6A1
ETFB
SOD2
PRDX3
Cortex
Human CSF
Mouse CSF
Serum CSF
TMT Intensity (Log2)
Ctl
AD

Supplementary Figure 4: **Mitochondrial proteins that have decreased expression levels in AD in all three analyzed biofluids**

Dot plots overlaid onto boxplots showing expression of proteins in human cortex, human CSF, mouse CSF and serum. X-axis shows sample groups, y-axis indicates Log_2 transformed TMT intensity. Boxplot center line, median; box limits, upper and lower quartiles; whiskers, 1.5x interquartile range; points, expression levels of each individual samples.
